# Preparation of Lignin Nanoparticles from *Thlaspi arvense* L. Rhizomes via Ultrasound-Assisted Antisolvent Precipitation: Nanostructural Characterization and Evaluation of Their Radical Scavenging Activity

**DOI:** 10.3390/molecules30204070

**Published:** 2025-10-13

**Authors:** Ru Zhao, Wenjun Xu, Yuxiang Tang, Jinwen Liu, Xiaoli Li, Liangui Tan, Ailing Ben, Tingli Liu, Lei Yang

**Affiliations:** 1 Nanjing Engineering Research Center for Peanut Genetic Engineering Breeding and Industrialization, School of Food Science, Nanjing Xiaozhuang University, Nanjing 211171, China; 2021044@njxzc.edu.cn (R.Z.);; 2 Key Laboratory of Forest Plant Ecology, College of Chemistry, Chemical Engineering and Resource Utilization, Ministry of Education, Northeast Forestry University, Harbin 150040, China

**Keywords:** ultrasound-assisted antisolvent precipitation, *Thlaspi arvense* L. rhizomes, lignin nanoparticles, solubility, radical scavenging activity

## Abstract

The ultrasound-assisted antisolvent precipitation method was used to prepare lignin nanoparticles from *Thlaspi arvense* L. rhizomes. The influence of each experimental variable on the average particle size (APS) of the lignin nanoparticles was determined via single-factor experiments. The optimal conditions for the preparation of the lignin nanoparticles were investigated in detail, and the APS of the lignin nanoparticles was 118 ± 3 nm. Compared with those of untreated lignin, the lignin nanoparticles prepared via this method were spherical and evenly distributed, and the structure was not damaged. Ultrasound generated local extreme physical conditions through its cavitation effect to promote nucleation, triggered high-speed turbulence to refine the particle size and improve uniformity, and applied mechanical disturbance to inhibit particle agglomeration, which promoted the preparation of lignin nanoparticles with a small size and good dispersion. A solubility test revealed that the lignin nanoparticles had greater solubility, which was improved 9-fold. The determination of antioxidant capacity revealed that the lignin nanoparticles had high free radical scavenging activity, which provided a broader space for the multifaceted utilization of a kind of grass lignin with the structural characteristics of *T. arvense* lignin (*p*-hydroxyphenyl lignin).

## 1. Introduction

With the rapid development of the global economy, nonrenewable resources such as coal, oil, and natural gas are continuously consumed, and fossil resources are increasingly being exhausted [1,2]. Therefore, the demand for sustainable and renewable resources is gradually increasing. The annual output of biomass renewable materials based on lignin is approximately 70 million tons, which mainly comes from the pulping waste liquid of the paper industry. However, only approximately 5% of lignin is used, and the remainder is burned or discharged into rivers as byproducts, causing severe pollution to the environment [3,4]. Lignin production will continue to increase over the next few years [5]. Therefore, realizing the effective utilization of lignin plays an important role in environmental protection and improving resource utilization.

Lignin is a natural phenolic polymer with reserves second only to those of cellulose. It is the only renewable aromatic compound with the richest content on earth and is a byproduct of the pulp and paper industry, which has the characteristics of environmental protection, sustainability, and biodegradability [6,7,8,9]. Lignin’s molecular structure features numerous reactive functional groups, including methoxy, phenolic hydroxyl, and carboxyl groups [10], which are endowed with antibacterial, antioxidant, and biocompatible properties [11]. In particular, lignin has good free radical scavenging ability, which can reduce the generation of oxygen free radicals and stabilize the oxidation reaction. However, the direct use of lignin, a high-molecular-weight polymer with high structural complexity, poor dispersion, and uneven molecular weight, easily leads to poor product stability and low value, and realizing the effective use of lignin is difficult [12,13]. At present, the main source of lignin separation in industry is from pulping waste liquid [14]. Owing to the long growth cycle of trees and the shortage of resources, the proportion of gramineous plants used as papermaking raw materials is increasing. Therefore, further treatment of lignins with *p*-hydroxyphenyl lignin (H-type) structures is highly important for the full utilization of herbaceous plant resources.

The preparation of macromolecular lignin into nanoparticles can effectively improve its utilization range [15,16]. Lignin nanoparticles are environmentally friendly materials with the advantages of a large specific surface area and high reactivity [15,17]. Other studies have shown that lignin nanoparticles can play a greater antioxidant role than non-nanolignin [11]. At present, lignin nanoparticles are used in drug delivery, UV shielding, antibacterial materials, food packaging materials, biobased fillers, biophoton materials, nerve tissue repair materials, and other fields [14,18,19,20,21,22,23]. The methods used to prepare lignin nanoparticles include the crosslinking method, mechanical method, biological method, and antisolvent precipitation method [24]. Among the various techniques for producing lignin nanoparticles, antisolvent precipitation stands out as the simplest, most efficient, and cost-effective approach. This method uses the amphiphilic nature of lignin molecules (hydrophilic groups such as hydroxyl and carboxyl groups and hydrophobic groups with benzene ring structures) to form spherical nanoparticles when mixing two or more solutions. Ultrasound is also used in antisolvent crystallization technology, mainly because the cavitation effect of ultrasound promotes the nucleation process and a uniform particle size distribution [25]. The fundamental principle of ultrasonic application in the preparation of nanoparticles by the antisolvent precipitation method is based on its cavitation effect [16]. When ultrasound waves penetrate a saturated solution, high temperatures, high pressures, and high cooling rates are generated when cavitation bubbles are formed and destroyed, which promotes the effective collision of molecules, the supersaturation of partial solutions, and then primary nucleation [26]. In addition, previous studies have shown that the breakdown of cavitation bubbles can trigger high-speed turbulence, which can effectively reduce the particle size of large-particle crystals [27]. Therefore, the particle size of lignin particles prepared by combining the traditional antisolvent method with ultrasonication can be relatively small.

*Thlaspi arvense* L., an annual herb in the Cruciferae family, is also a winter oil crop that is widely distributed in temperate North America and Eurasia [28,29]. As a winter mulching crop worthy of attention, *T. arvense* has low agricultural investment and an early harvest, which makes it suitable for planting in conventional corn–soybean rotations in the Midwest United States. Therefore, it does not replace existing food crops, nor does it need additional land for planting, but it also provides economic benefits and makes the ecosystem more diversified [28,30,31]. In addition, as a traditional medicinal plant in China, penniselle is widely distributed throughout China. Because penniselle is often regarded as a field weed, its application value is ignored, resulting in a waste of resources and potential economic losses [32]. The main components of the whole plant of *T. arvense* include the seeds, leaves, and rhizomes of *T. arvense*. Since ancient times, research on *T. arvense* has focused mostly on seeds and leaves, while the utilization of rhizomes has been relatively rare. The seeds of *T. arvense* are rich in seed oil (20%-36%) and have a high erucic acid content. It is mainly used in industry to prepare soap, lubricating oil, and biodiesel with high oil contents [32,33]. The essential oil of *T. arvense* has antifungal activity against *Penicillium expansum* [34]. The rhizomes of *T. arvense* are similar to those of straw and are often wasted as a byproduct of crops and are rich in lignin resources. The extraction and utilization of lignin from the rhizomes of *T. arvense* will be highly important for improving the comprehensive utilization of *T. arvense* resources.

In this work, the rhizomes of *T. arvense* were first used as raw materials to prepare lignin nanoparticles via an ultrasound-assisted antisolvent precipitation method. A single-factor experiment was used to determine the change trend of the influence of each experimental variable on the average particle size (APS) of the lignin nacnoparticles. The Plackett–Burman and Box–Behnken designs were used to optimize the conditions for the preparation of lignin nanoparticles, and a validation test was carried out to determine the optimal conditions for the preparation of lignin nanoparticles from *T. arvense* rhizomes. The lignin nanoparticles prepared via the ultrasound-assisted antisolvent method were characterized, and the solubilities and antioxidant capacities of the prepared lignin nanoparticles were determined, which provided a broader space for the comprehensive utilization of *T. arvense* and the utilization of the plant lignin represented by *T. arvense* lignin.

## 2. Results and Discussion

### 2.1. Single-Factor Experiments

#### 2.1.1. Effect of the Dropping Speed on the Average Particle Size of Lignin Nanoparticles

**Figure 1:**
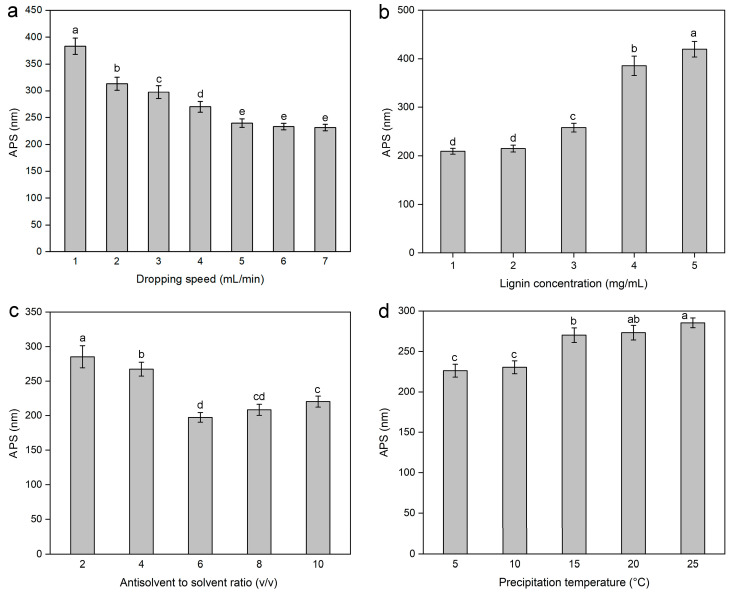
Effect of the dropping speed (**a**), lignin concentration (**b**), antisolvent-to-solvent ratio (**c**), and precipitation temperature (**d**) on the average particle size (APS) of lignin nanoparticles. Values with different letters are significantly different (*p* < 0.05).

The dropping speed affects the amount of sample in contact with the antisolvent per unit time, thereby influencing the number of nuclei of the sample in the antisolvent. Therefore, the effects of different dropping speeds (1, 2, 3, 4, 5, 6, and 7 mL/min) on the APS of the lignin nanoparticles were investigated while keeping other conditions constant, specifically, a lignin concentration of 3 mg/mL, an antisolvent-to-solvent volume ratio of 4:1 (*v/v*), a precipitation temperature of 15 °C, a stirring speed of 900 r/min, and an ultrasound irradiation and time of 100 W for 5 min. The results are shown in Figure 1a. As the dropping speed increased from 1–7 mL/min, the APS of the lignin nanoparticles concentration first decreased gradually and then tended to level off. This occurred because the growth rate of the crystals depends on the dropping speed. At lower dropping speeds, the solvent/antisolvent mixture per unit time was small, and there were almost no nucleation sites, which prolonged the crystal growth process and led to the formation of larger crystals. However, as the flow rate increased, the solvent/antisolvent mixture amount per unit time increased, so the nucleation amount of lignin nanoparticles gradually increased. Owing to the very short time available for nucleus growth, only small individual nuclei were formed [35]. Therefore, the range of 4–6 mL/min was selected as the appropriate dropping speed.

#### 2.1.2. Effect of Lignin Concentration on the Average Particle Size of Lignin Nanoparticles

The lignin concentration affects the lignin viscosity to a certain extent, thereby exerting a significant effect on the APS of the prepared lignin nanoparticles. Therefore, the effects of different lignin concentrations (1, 2, 3, 4, and 5 mg/mL) on the APS of the lignin nanoparticles were investigated, with other conditions held constant, namely, a dropping speed of 4 mL/min, antisolvent-to-solvent volume ratio of 4:1 (*v/v*), precipitation temperature of 15 °C, stirring speed of 900 r/min, and ultrasound irradiation and time at 100 W for 5 min. As shown in Figure 1b, the APS of the lignin nanoparticles gradually increased with increasing lignin concentration. This phenomenon could be explained from the following two aspects: the nucleation rate at the solvent-antisolvent interface and the influence of the lignin concentration on the solution viscosity [36]. First, higher lignin concentrations resulted in the formation of many nuclei at the two-phase interface, leading to the aggregation of nuclei, which, in turn, resulted in the formation of larger lignin nanoparticles. At lower concentrations, lignin saturation and precipitation processes occurred relatively late during droplet spreading. Thus, the primary mechanism for producing smaller particles was nucleation. With increasing concentration, the solution reached saturation earlier, causing the growth process to coincide with nucleation, thereby extending the time available for crystal growth. Therefore, the overlapping growth of crystal nuclei was the main reason for the formation of larger particles [37]. In addition, higher concentrations increased the viscosity and surface tension of the solution, stabilizing the jet flow and promoting larger primary droplets, ultimately leading to the formation of larger particles. The increase in lignin viscosity hindered the diffusion of the lignin solution in the antisolvent [38,39]. Furthermore, the inhomogeneous supersaturated solution led to the formation of larger particles. Therefore, the lignin concentration range of 1–3 mg/mL was used in the experiment to screen for significant factors in PBD.

#### 2.1.3. Effects of the Antisolvent-to-Solvent Ratio on the Average Particle Size of Lignin Nanoparticles

The volume ratio of antisolvent to solvent is a key factor affecting the APS of lignin nanoparticles. The effects of different antisolvent-to-solvent ratios (2, 4, 6, 8, and 10 *v/v*) on the APS of the lignin nanoparticles were investigated, with the dropping speed maintained at 4 mL/min, the lignin concentration at 3 mg/mL, the precipitation temperature at 15 °C, the stirring speed at 900 r/min, and the ultrasound irradiation power and time at 100 W for 5 min. The results are shown in Figure 1c. As the antisolvent-to-solvent ratio increased from 2:1 to 6:1 and then to 10:1, the APS of the lignin nanoparticles ratio first decreased but then gradually increased. During the precipitation process, once nuclei are formed, growth occurs simultaneously. For subsequent growth, a higher volume ratio of antisolvent to solvent increases the diffusion distance of growth substances. Thus, diffusion becomes a limiting factor for nucleus growth, which, in turn, reduces the particle size. This may explain why the particle size of nanolignin decreases within the range of antisolvent-to-solvent volume ratios from 2:1 to 4:1 (*v*/*v*). However, when the volume ratio of antisolvent to solvent continues to increase, the amount of lignin in contact with the antisolvent per unit time remains unchanged. Therefore, a higher antisolvent-to-solvent ratio, in contrast, increases the distance for the uniform spread of lignin into the antisolvent, which may be the reason for the increase in the APS of the lignin nanoparticles. Considering the influence of the antisolvent-to-solvent ratio on the APS of the lignin nanoparticles and the need to avoid solvent waste, an antisolvent to solvent ratio (*v*/*v*) ranging from 6–8 was selected for the subsequent screening experiment of significant factors in PBD.

#### 2.1.4. Effect of Precipitation Temperature on the Average Particle Size of Lignin Nanoparticles

The effects of different precipitation temperatures (5, 10, 15, 20, and 25 °C) on the APS of the lignin nanoparticles were investigated, with the dropping speed maintained at 4 mL/min, the lignin concentration at 3 mg/mL, the antisolvent-solvent ratio at 4:1 (*v*/*v*), the stirring speed at 900 r/min, and the ultrasound irradiation power and time at 100 W for 5 min. The result is shown in Figure 1d. As shown in the figure, lower temperatures could produce lignin nanoparticles with smaller particle sizes. This may be because lower temperatures can prevent aggregation during the nucleation process, thus resulting in the formation of smaller lignin particles. The figure also showed that selecting a precipitation temperature of 10 °C can maintain a relatively small APS of the lignin nanoparticles.

#### 2.1.5. Effect of Stirring Speed on the Average Particle Size of Lignin Nanoparticles

**Figure 2:**
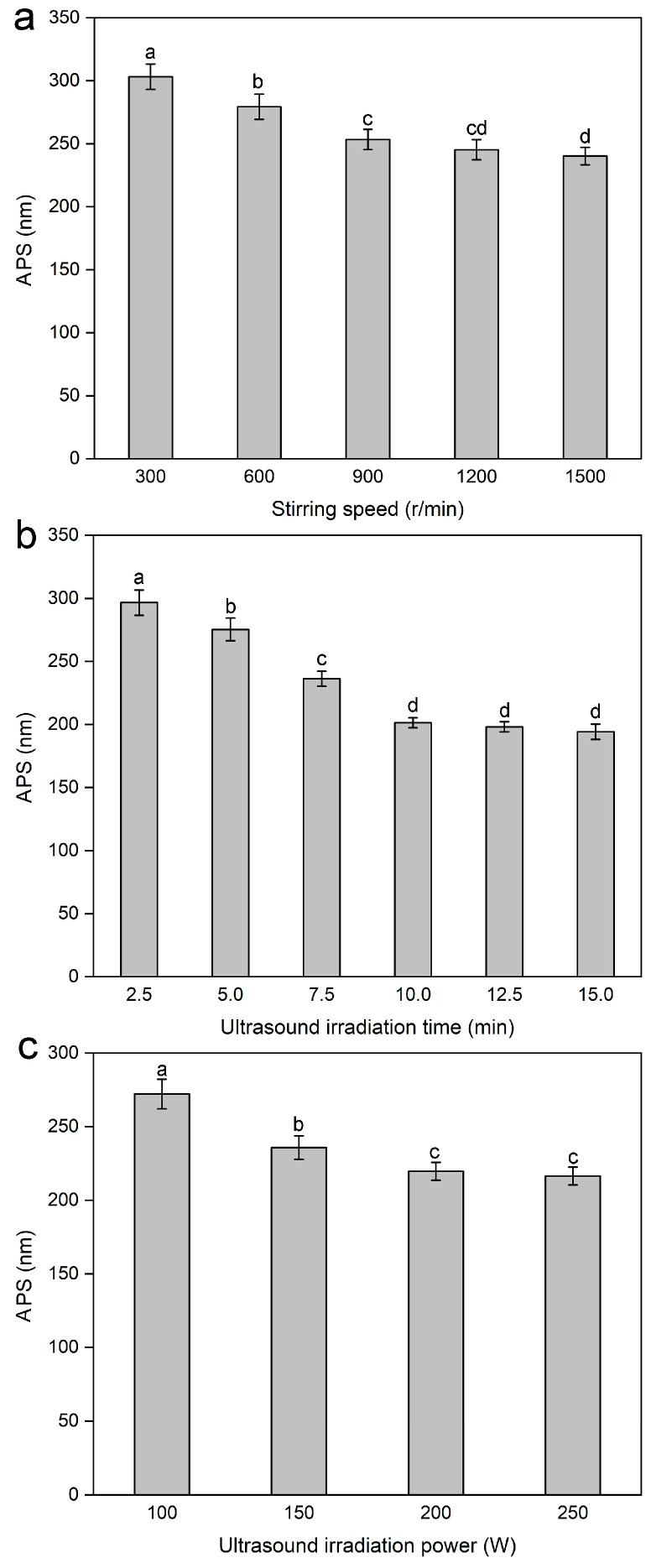
Effect of stirring speed (**a**), ultrasound irradiation time (**b**), and ultrasound irradiation power (**c**) on the average particle size (APS) of the lignin nanoparticles. Values with different letters are significantly different (*p* < 0.05).

The stirring speed plays an irreplaceable role in the formation process of lignin nanoparticles, thereby affecting the APS of lignin nanoparticles. As shown in Figure 2a, as the stirring speed increased to 900 r/min, the APS of the lignin nanoparticles decreased significantly; however, when the stirring speed continued to increase, the decrease in the APS of the lignin nanoparticles became insignificant. This phenomenon indicated that an appropriate stirring speed was conducive to the uniform distribution of the lignin solution in the antisolvent, which, in turn, facilitated the formation of smaller lignin particles. Furthermore, the decrease in the APS of the lignin nanoparticles with increasing stirring speed was due to the increase in micromixing motion between multiple phases (mixing at the molecular level) [40]. A high micromixing efficiency improved the mass transfer and diffusion rates between multiple phases, which caused high homogeneous supersaturation in a short time, thereby promoting rapid nucleation to generate smaller lignin particles. Therefore, a relatively high stirring speed was beneficial for the formation of smaller and more uniform lignin nanoparticles, and the range of 600–1200 r/min was selected for the screening experiment of significant factors in PBD.

#### 2.1.6. Effect of Ultrasound Irradiation Time on the Average Particle Size of Lignin Nanoparticles

The ultrasonic process helps prevent aggregation between particles, resulting in particles of a uniform size, and the ultrasound irradiation time is a key factor in obtaining uniform and small-sized lignin particles. Therefore, the effects of different ultrasound irradiation times (2.5, 5, 7.5, 10, 12.5, and 15 min) on the APS of the lignin nanoparticles were studied, with the dropping speed maintained at 4 mL/min, the lignin concentration at 3 mg/mL, the antisolvent-to-solvent volume ratio at 4:1 (*v*/*v*), the precipitation temperature at 15 °C, the stirring speed at 900 r/min, and the ultrasound irradiation power at 100 W unchanged. As shown in Figure 2b, when the ultrasound irradiation time was within 10 min, the APS of the lignin nanoparticles gradually decreased with increasing time. However, when it exceeded 10 min, the APS of the lignin nanoparticles did not decrease significantly. Thus, an ultrasound irradiation time of 10 min could ensure the formation of lignin nanoparticles with uniform and relatively small particle sizes, and an ultrasound irradiation time ranging from 7.5–12.5 min was selected for screening the significant factors in the PBD.

#### 2.1.7. Effect of Ultrasound Irradiation Power on the Average Particle Size of Lignin Nanoparticles

Ultrasound treatment causes the high-frequency vibration of lignin, thereby reducing the size of large lignin particles and obtaining lignin nanoparticles. Ultrasound irradiation power is the driving force for the uniform diffusion of lignin into the antisolvent. Therefore, the effects of different microwave irradiation powers (100, 150, 200, and 250 W) on the APS of the lignin nanoparticles were investigated, with the dropping speed maintained at 4 mL/min, the lignin concentration at 3 mg/mL, the antisolvent-to-solvent volume ratio at 4:1 (*v*/*v*), the precipitation temperature at 15 °C, the stirring speed at 900 r/min, and the ultrasound irradiation time at 5 min unchanged. As shown in Figure 2c, a higher power made it easier to form lignin with smaller particles. The ultrasound irradiation power significantly affects cavitation by affecting compression and rarefaction cycles; the higher the ultrasound irradiation power is, the greater the number of cavities [41]. Owing to the action of shock waves and liquid splashing, higher ultrasonic irradiation power can accelerate the diffusion of the lignin solution in the antisolvent and prevent the aggregation of the formed lignin nanoparticles, thus ensuring the formation of uniform lignin nanoparticles with smaller particle sizes. Therefore, the microwave irradiation power range of 150–250 W was selected as a candidate for screening for significant factors in PBD.

### 2.2. Screening of Significant Factors Affecting Lignin Nanoparticle Preparation via PBD

PBD can evaluate the influence of factors on response values through a relatively small number of experiments. Therefore, to reduce the complexity of the experiments, PBD was used to assess the degree of influence of parameters on the APS of the lignin nanoparticles and screen out the factors with significant influence, laying a foundation for subsequent optimization.

**Figure 3:**
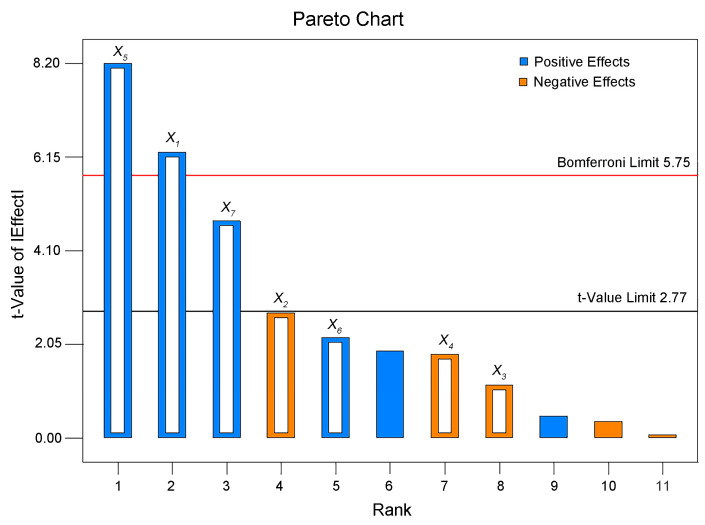
Pareto chart of the effects of seven variables on the preparation process of lignin nanoparticles.

On the basis of the experimental results in Section 2.1, the optimization ranges of seven parameters were selected for PBD screening. The different combinations of PBD variables and the APS of the lignin nanoparticles are shown in Table 1. The Pareto chart drawn according to the PBD experimental results is presented in Figure 3. In the Pareto chart, the length of each column is proportional to the absolute value of the t statistic of the influencing factor, reflecting the degree of influence of the factor on the response value. As shown in the figure, the t limit value was 2.77, and the Bonferroni limit value was 5.75. By comparing the t values of different factors with these limits, it could be concluded that a factor has a significant influence on the response value when its t value was greater than the t limit value and an extremely significant influence when its value was greater than the Bonferroni limit value. Therefore, the dropping speed (*X*_1_) and stirring speed (*X*_5_) are extremely significant factors, and the ultrasound irradiation power (*X*_7_) is a significant factor.

The significant factors affecting the APS of the lignin nanoparticles were screened and predicted through analysis of variance and regression analysis of the PBD data, and the results are shown in Table 2. As indicated in the table, the dropping speed (*X*_1_), stirring speed (*X*_5_), and ultrasound irradiation power (*X*_7_) are all factors with significant influences, with *p*-values of 0.0033, 0.0012, and 0.0090, respectively. The order of significance of the seven factors affecting the APS of the lignin nanoparticles is as follows: dropping speed (*X*_1_) > stirring speed (*X*_5_) > ultrasound irradiation power (*X*_7_) > lignin concentration (*X*_2_) > ultrasound irradiation time (*X*_6_) > precipitation temperature (*X*_4_) > antisolvent-to-solvent ratio (*X*_3_). Compared with the t values, the influences of the lignin concentration (*X*_2_), ultrasound irradiation time (*X*_6_), precipitation temperature (*X*_4_), and antisolvent-to-solvent ratio (*X*_3_) on the APS of the lignin nanoparticles were relatively weak. Among them, the lignin concentration, antisolvent-to-solvent ratio, and precipitation temperature had positive effects on the APS of the lignin nanoparticles, whereas the other four factors had negative effects. A smaller APS of the lignin nanoparticles was more conducive to efficient utilization. Based on the single-factor and PBD results, the lignin concentration was determined to be 1 mg/mL, the antisolvent-to-solvent ratio was 6:1, the ultrasound irradiation time was 10 min, and the precipitation temperature was 10 °C. The other three factors were further determined after optimization via BBD.

### 2.3. Optimization of the Optimal Conditions for Lignin Nanoparticle Preparation via BBD

#### 2.3.1. Model Establishment and Analysis

Taking the APS of the lignin nanoparticles as the response value, the 17 combinations of actual values of the three factors (*X*_1_: dropping speed, *X*_5_: stirring speed, *X*_7_: ultrasound irradiation power) and their corresponding results are shown in Table 3, and Table 4 displays the BBD-derived response surface quadratic model’s regression coefficients and ANOVA results for the APS of the lignin nanoparticles (*Y*) determined via BBD. A multiple regression equation for the APS of the lignin nanoparticles (*Y*) was obtained, as shown in the following equation:

Y = 691.77 − 62.26 X_1_ − 0.21 X_5_ – 2.29 X_7_ − 3.92 × 10^−3^ X_1_X_5_ + 0.06 X_1_X_7_ + 5.33 × 10^−5^ X_5_X_7_ + 3.36 X_1_^2^ + 7.84 × 10^−5^ X_5_^2^ + 3.97 × 10^−3^ X_7_^2^

The correlation coefficient (*R*^2^) for the APS of the lignin nanoparticles is 0.9966, and the adjusted determination coefficient (Adj. *R*^2^) of the prediction model is 0.9923, which indicates a high correlation between the actual and predicted values of the APS of the lignin nanoparticles. After statistical analysis, the *F*-value of the APS of the lignin nanoparticles model is 230.57, all of which are highly significant (*p*-value < 0.0001), and the *p*-value of the lack-of-fit term is not significant (*p* > 0.05), suggesting that the model is accurate and reliable. The coefficient of variation (CV) represents the reproducibility of the model, and the CV of the APS of the lignin nanoparticles is 1.09%, indicating that the experimental model has high accuracy. Adequate precision is used to determine the signal-to-noise ratio; the adequate precision of the APS of the lignin nanoparticles model is 47.845, which is greater than 4, indicating that there is a sufficient signal for the experiment.

#### 2.3.2. Analysis of Response Surface Plots

**Figure 4:**
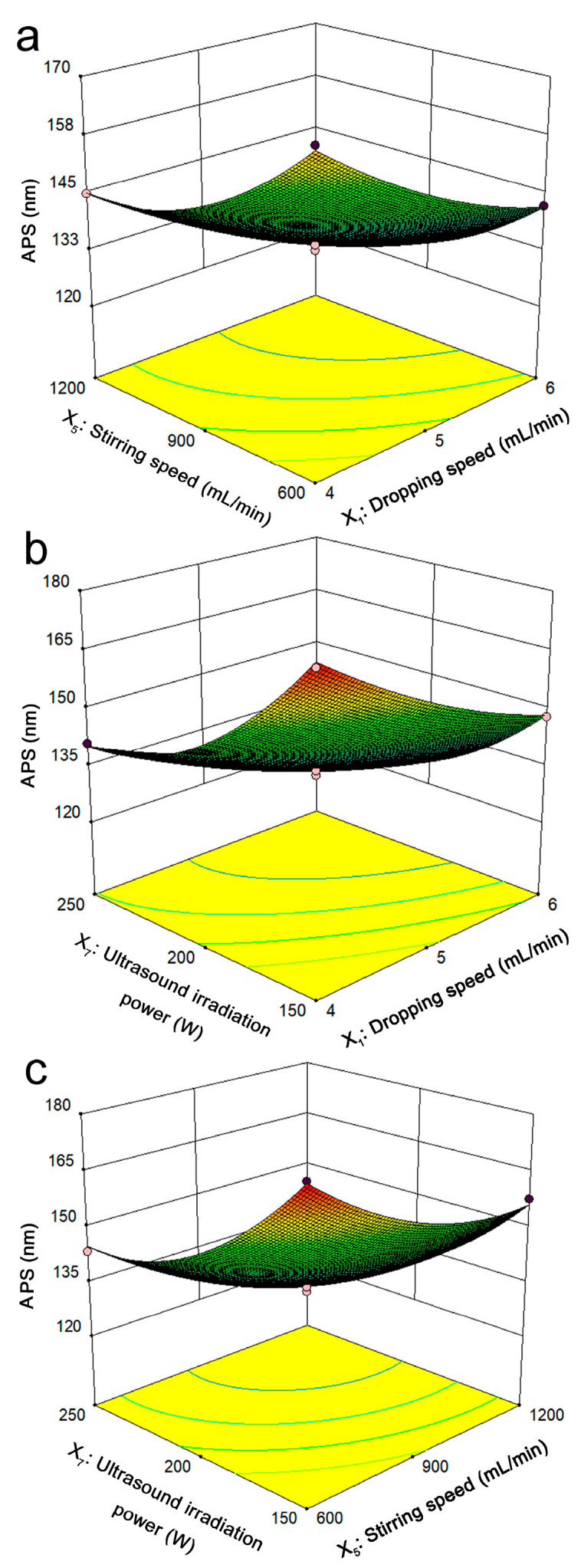
Optimization of the average particle size (APS) of the lignin nanoparticles via Box–Behnken design (BBD). Interaction effect of the dropping speed and stirring speed on the APS of the lignin nanoparticles (**a**); interaction effect of the dropping speed and ultrasound irradiation power on the APS of the lignin nanoparticles (**b**); interaction effect of the stirring speed and ultrasound irradiation power on the APS of the lignin nanoparticles (**c**).

The interaction effects of the three factors (dropping speed, stirring speed, and ultrasound irradiation power) on the size of the APS of the lignin nanoparticles can be intuitively reflected by three-dimensional response surface plots. One of the variables was controlled at the 0 level, with the APS of the lignin nanoparticles as the *Z*-axis, and the remaining two variables were controlled at the *X*-axis and *Y*-axis to draw 3D response surface plots, as shown in Figure 4a–c.

The interaction effect of the lignin dropping speed and stirring speed on the size of the APS of the lignin nanoparticles is shown in Figure 4a, where the ultrasound irradiation power was fixed at 200 W. As the drop rate and stirring speed increased, the APS of the lignin nanoparticles decreased significantly and then tended to be stable. The effects of the lignin drop rate, ultrasonic irradiation power, and stirring speed on the size of the APS of lignin nanoparticles are shown in Figure 4b,c, respectively. The figures show that the APS of the lignin nanoparticles was significantly smaller under ultrasonic action, indicating that the ultrasonic-assisted antisolvent method for preparing lignin nanoparticles was beneficial for the formation of lignin particles with smaller particle sizes.

#### 2.3.3. Verification Experiments

To evaluate the validity and accuracy of the prediction model and results, three replicate experiments were conducted under the optimal conditions derived from the response surface analysis. The optimized conditions for the minimum APS of the lignin nanoparticles obtained through BBD experiments were as follows: dropping speed of 6 mL/min, stirring speed of 1145.35 r/min, and ultrasound irradiation power of 246.97 W. Considering both the APS of the lignin nanoparticles and the convenience of operation in actual experiments, the actual parameter conditions were slightly modified as follows: dropping speed of 6 mL/min, stirring speed of 1150 r/min, and ultrasound irradiation power of 247 W. Under these conditions, three verification experiments were carried out. The size of the prepared APS of the lignin nanoparticles was 118 ± 3 nm, which is very close to the predicted value of 119 nm, indicating that the selected BBD optimization conditions are reliable.

### 2.4. Model Adequacy Survey

Model adequacy was measured by some diagnostic plots. Three diagnostic plots, namely, the actual responses versus the predicted responses, the normal plot of residuals and internally Studentized residuals versus run number, are shown in Figure S1. Figure S1a shows that all reasonably aligned points were distributed and rotated around a straight line, which showed a high degree of matching between the actual and predicted values obtained by the model [42]. As we can see from Figure S1b, all points in the normal plot of residuals were close to contact with the straight line, meaning that the model of the lignin nanoparticles was stable and accurate and complied with a normal distribution. The plot of internally Studentized residuals versus run number was employed, as shown in Figure S1c. All data points exhibited a random scatter distribution within certain limits (±3), which showed a good fit of the responses to the developed model. 

### 2.5. Characterization of Lignin Nanoparticles

#### 2.5.1. Scanning Electron Microscopy

**Figure 5:**
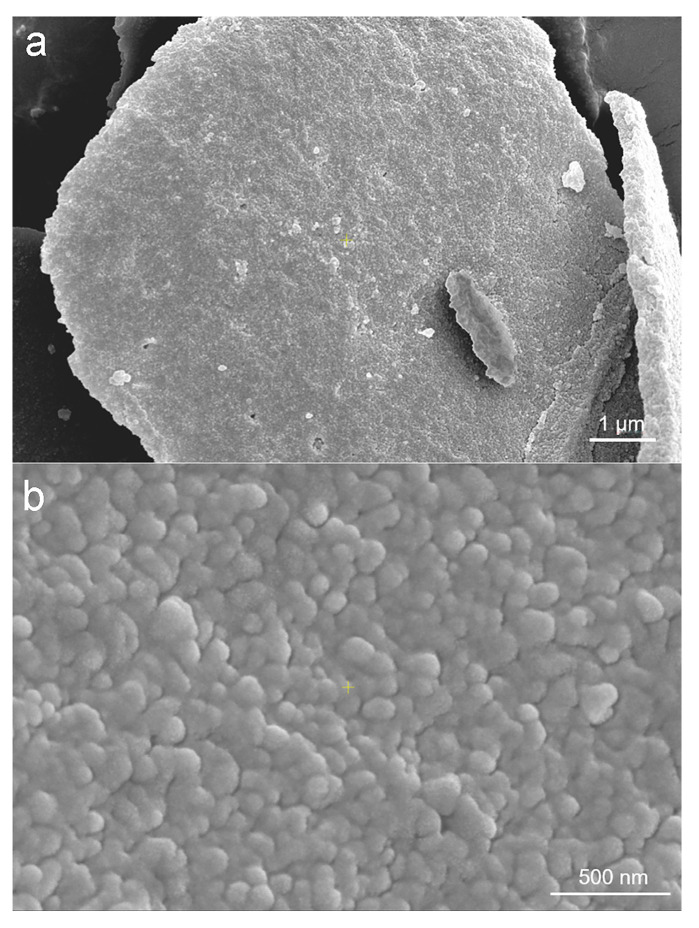
SEM images of non-nanosized lignin (**a**) and lignin nanoparticles (**b**).

The morphologies of the untreated lignin and lignin nanoparticles prepared via the ultrasonic-assisted antisolvent method were compared via SEM, and the results are shown in Figure 5. As shown in Figure 5a, the untreated lignin exhibited obvious aggregation and irregular bulk structures, with an uneven distribution. In contrast, the lignin prepared by the ultrasound-assisted antisolvent method appeared spherical, was uniformly distributed, and did not aggregate into blocks, as shown in Figure 5b. Moreover, the particle size of the lignin prepared after ultrasonic treatment was significantly smaller than that of the untreated lignin.

#### 2.5.2. FTIR Analysis

**Figure 6:**
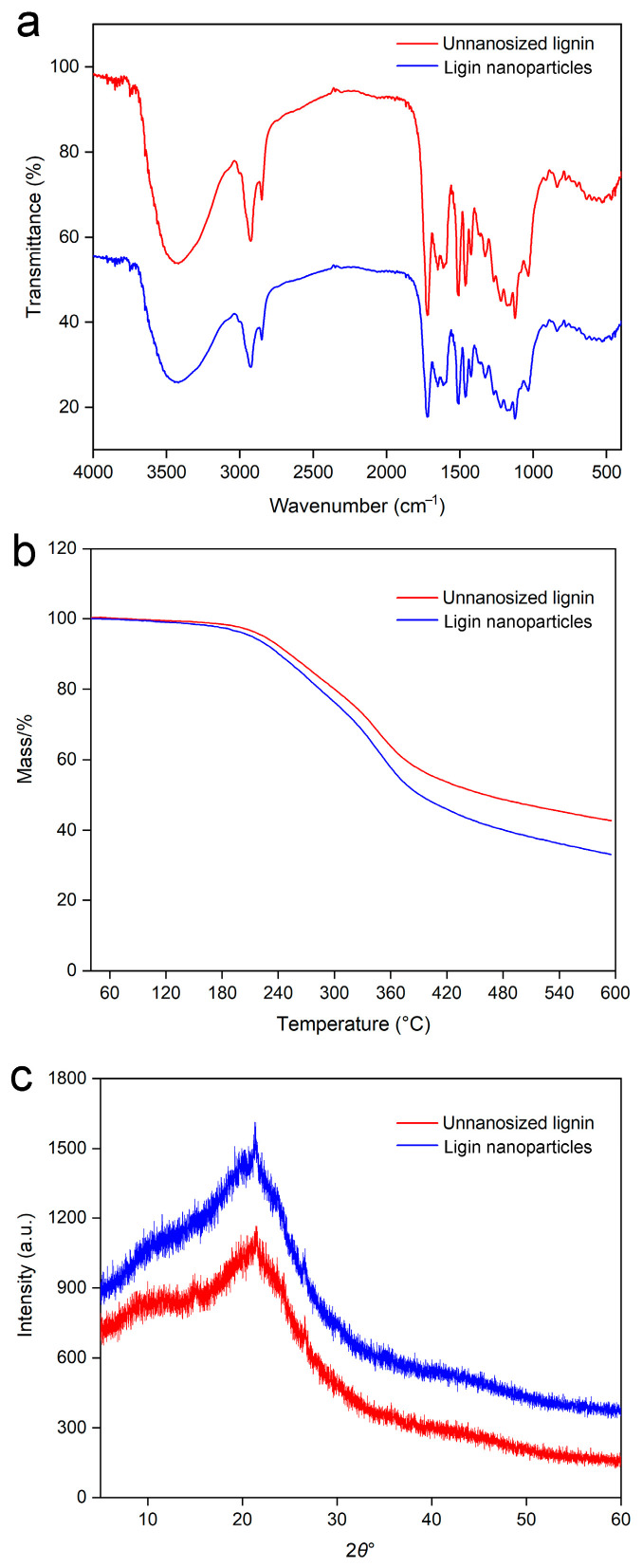
FTIR (**a**), TG (**b**), and XRD (**c**) images of non-nanosized lignin and lignin nanoparticles.

The infrared spectra of the lignin before and after nanonization are shown in Figure 6a. The figure shows that the vibration peaks of the lignin nanoparticles and the untreated lignin were similar, indicating that the structure of the lignin nanoparticles prepared by this method was not destroyed.

#### 2.5.3. Thermogravimetric Analysis

Thermogravimetry (TG) measurements were performed on the samples to evaluate the thermal degradation behavior of lignin, which usually ranges from 185–112 °C because of the complex structure of phenolic hydroxyl groups and carbonyl and benzyl hydroxyl groups in lignin [43]. The thermogravimetric curves of the lignin nanoparticles prepared via the ultrasound-assisted antisolvent method and the untreated lignin are shown in Figure 6b, from which the TG curve could be divided into three stages. In the first stage of 40–150 °C, the two samples has similar weight losses; the second stage corresponded to 150–380 °C; and in the third stage of 380–600 °C, the mass loss of the lignin nanoparticles was greater than that of the untreated lignin, which was caused by the smaller particle size and specific surface area of the lignin prepared by the ultrasound-assisted antisolvent method. The decomposition of lignin structures was reported to occur at relatively low temperatures (150–290 °C) [44], which was consistent with the phenomenon observed in this study. The initial weight loss resulted from hydroxyl group dehydration in the benzyl moieties. Cleavage of *α*- and *β*-aryl-alkyl ether bonds has been reported to occur at approximately 150 and 300 °C [45]. At approximately 300 °C, aliphatic side chains began to separate from aromatic rings, whereas at 370–400 °C, C-C cleavage occurred between lignin building blocks [46]. Finally, at temperatures above 500 °C, the weight loss of TG tended to flatten, and some volatile products, such as CO, CO_2_, CH_4_, and H_2_, formed during lignin pyrolysis, were slowly released before carbon (30–50%) was formed [47].

#### 2.5.4. X-Ray Diffraction Analysis

The XRD analysis of lignin before and after nanonization is shown in Figure 6c. The figure shows that the lignin both before and after treatment by the ultrasonic-assisted antisolvent method exhibited an amorphous state. However, the diffraction peak intensity of the lignin nanoparticles prepared by the ultrasound-assisted antisolvent method was lower than that of the untreated lignin. This was attributed to the particle size of the lignin, which also indicated that the ultrasound-assisted antisolvent method was conducive to the formation of lignin with smaller particles.

### 2.6. Solubility Determination of Lignin Samples

**Figure 7:**
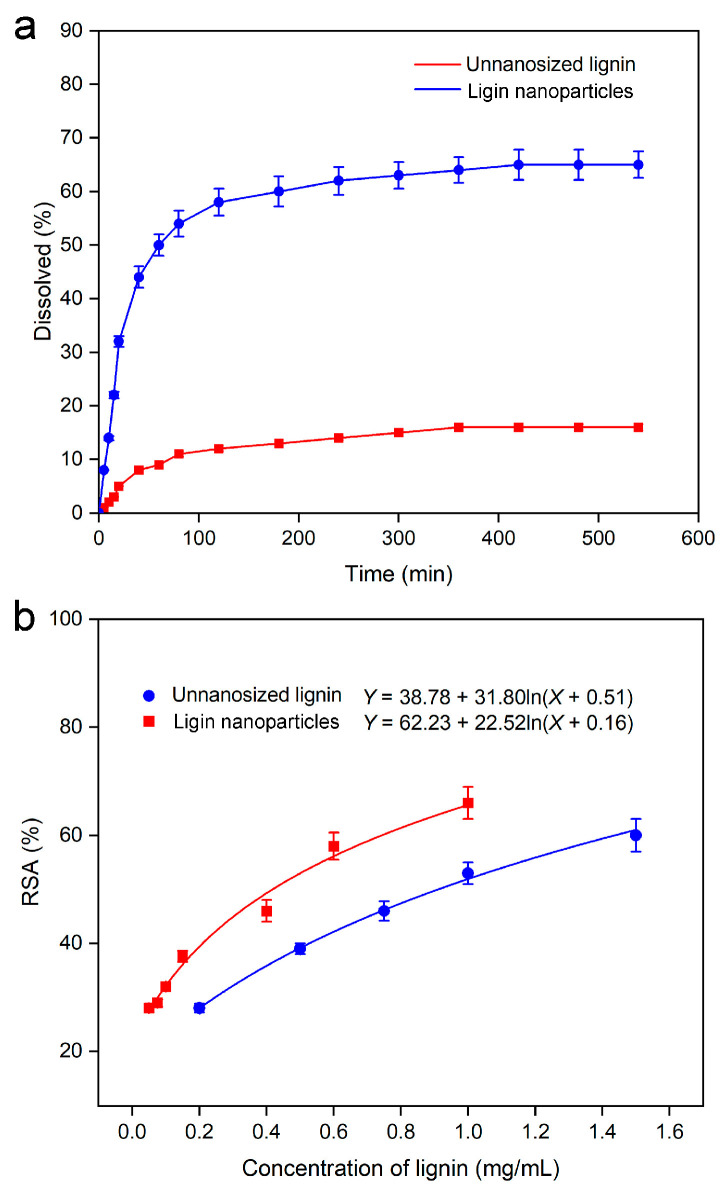
Solubility (**a**) and radical scavenging activity (RSA%) analysis (**b**).

The solubility curves of the lignin samples are shown in Figure 7a. The figure shows that the solubility of the lignin nanoparticles was over 60% and greatly improved and was approximately 9 times greater than the solubility of the non-nanosized lignin samples, indicating that the lignin nanoparticles had a better dissolution effect than the untreated lignin. Amorphous materials exhibited higher energy states than their crystalline counterparts. When the sample existed in an amorphous state, too much free energy and entropy was generated in the solid, because the rapid solidification made the basic structural unit unable to reach its minimum energy state. This inherent thermodynamic instability consequently enhanced the solubility [48].

Tran et al. identified two key factors enhancing sample dissolution: the transition to an amorphous state and particle size reduction, both of which improve wettability [49]. Reducing the particle size increased the sample bioavailability through improved dissolution, whereas the crystalline/amorphous state significantly influenced the solubility and bioavailability. Therefore, lignin nanoparticles prepared by ultrasound-assisted antisolvent extraction have increased solubility, which provides a foundation for the development and utilization of lignin nanoparticles from the rhizome of *T. arvense.*

### 2.7. Evaluation of the Radical Scavenging Activity of Lignin Samples

Lignin effectively scavenges free radicals through its phenolic hydroxyl groups, mitigating oxygen radicals and stabilizing oxidative processes [50,51]. Non-nanosized lignin and lignin nanoparticles samples were dissolved in aqueous solutions, prepared and diluted into solutions of different concentrations, and their free radical scavenging activities were tested, and the results are shown in Figure 7b, which presents different degrees of free radical scavenging activity, and the free radical scavenging activity was positively correlated with the sample concentration. The *IC*_50_ of the non-nanosized lignin and lignin nanoparticles samples was 0.41 ± 0.02 mg/mL and 0.91 ± 0.04 mg/mL, respectively. The DPPH of the free radical scavenging activity of the lignin nanoparticles was greater than that of the other samples, which was attributed to the fact that lignin nanoparticles offer significant advantages in comparison to conventional lignin powder, including enhanced surface activity, specific surface area, and other nanoscale properties, which facilitate better dispersion and more exposure of phenolic hydroxyl groups in aqueous solutions [52], thus improving the antioxidant activity of nanosized lignin. This provides a broader space for the multiple uses of a class of grass lignin characterized by lignin from *T. arvense*. Additionally, research has shown that lignin-based nanoparticles exhibit antioxidant and antibacterial properties, along with high cellular compatibility, making them suitable for tissue engineering applications [53].

### 2.8. Analysis of the Possible Mechanism of Ultrasound in the Preparation of Lignin Nanoparticles

In the process of preparing lignin nanoparticles by ultrasonic assisted antisolvent precipitation method, ultrasound plays a key role in the formation, size control, and distribution uniformity of nanoparticles through its unique cavitation effect and derivative effect. Its core mechanism may be divided into the following aspects: The core role of ultrasound is due to its cavitation effect: when ultrasound passes through the mixed system of lignin solution and antisolvent, a large number of small cavitation bubbles will be formed periodically [26]. When these cavitation bubbles burst instantaneously, extreme high temperatures, high pressures, and extremely high cooling rates will be generated locally. This violent physical environment can significantly promote the effective collision between lignin molecules, accelerate the local solution to reach a supersaturated state, trigger and enhance the primary nucleation process, and provide more initial nuclear sites for the formation of nanoparticles [26,54]. Secondly, the energy released when the cavitation bubbles burst will lead to high-speed turbulence and micro-jets. This strong hydrodynamic effect can effectively break up the possible large particle aggregates and reduce the average particle size of lignin nanoparticles; on the other hand, it can enhance the diffusion and mixing efficiency of the lignin solution in the antisolvent, avoid abnormal particle growth caused by uneven local concentration, and improve the particle size uniformity of the nanoparticles [25,27].

In addition, the continuous effect of ultrasound can also inhibit the agglomeration of particles: during the formation of nanoparticles, ultrasonic vibration can hinder the van der Waals force or hydrogen bond interaction between particles through mechanical disturbance, reduce the aggregation between newly formed nuclei, and further ensure the dispersion of products [54]. The experimental results also confirmed that appropriate ultrasonic power and time can significantly reduce the average particle size of lignin nanoparticles (118 ± 3 nm) and make a more uniform spherical distribution, which is directly related to the nucleation promotion, particle size refinement, and dispersion enhancement induced by the ultrasonic cavitation effect. Therefore, ultrasound plays an important role in the preparation of lignin nanoparticles with a small particle size and high dispersion, which lays the foundation for improving the solubility and free radical scavenging ability of lignin nanoparticles.

## 3. Conclusions

Lignin is often discarded as waste because of its color in industry, which not only leads to the waste of lignin resources but also causes serious environmental pollution due to the emission or combustion of a large amount of lignin. In this work, an ultrasound-assisted antisolvent precipitation approach was used to prepare lignin nanoparticles from *Thlaspi arvense* L. rhizomes with a uniform texture and small particle size. The optimal conditions for the preparation of lignin nanoparticles from *T. arvense* L. rhizomes were obtained via single-factor, PBD, and BBD optimization experiments. Under these conditions, the particle size of the lignin nanoparticles was 118 ± 3 nm. The solubility and antioxidation results revealed that lignin nanoions had increased solubility and free radical scavenging ability. However, there are also some issues worth noting and exploring, mainly including the following aspects: (1) The cost-effectiveness of this method: Compared with acid precipitation or supercritical antisolvent methods, ultrasound-assisted antisolvent precipitation involves organic reagents in the process of the ultrasonic method for preparing LNP and has the advantages of easy operation, low cost, and high efficiency. Therefore, the ultrasound-assisted antisolvent precipitation method for preparation of lignin nanoparticles also has relative advantages [25,55]. (2) The solvent environmental friendliness of this method: From the perspective of equipment and environmental protection, it is indeed necessary to use as few organic solvents as possible when conducting expansion experiments or factory applications. However, the issue of the particle size of lignin nanoions being limited by specific organic solvents still needs further exploration. (3) Limitations of research methods and processes: We did not consider that in actual industrial processes, parameters such as the lignin concentration, antisolvent ratio, temperature, and ultrasonic power may coexist and interact with each other. Therefore, when multiple factors change simultaneously, the trend observed under single-factor conditions may not fully reflect the actual system behavior. The possible interactions between multiple factors will be a focus of our future experiments. The exploration and resolution of the above issues in the future will contribute to the effective utilization of the lignin resources of *T. arvense* L. rhizomes and the application of lignin nanoparticles in medicine and food preservation. (4) The size stability of the nanoparticles obtained, the size distribution of lignin nanopar-ticles obtained by this method is relatively uniform and stable, and the size(118 ± 3 nm) is smaller compared to lignin nanoparticles obtained by method of supercritical antisolvent process (144 ± 3 nm) [55]. However, due to our current exploration and research at the la-boratory level, and considering the pursuit of cleaner and lower cost solvents, long-term stability experiments have not been reflected.

## 4. Materials and Methods

### 4.1. Raw Materials and Reagents

*T. arvense* rhizomes were purchased from the Anguo Traditional Chinese Medicine Market in Hebei Province. The samples were crushed via a grinder, sieved, and stored in a sealed and dry place in a fresh-keeping bag. The same batch of crushed raw materials was used for all the experiments in this study. The experimental reagents, including *n*-butanol, tetrahydrofuran, isopropanol, cyclohexane, dichloromethane, acetone, dimethyl sulfoxide (DMSO), ethyl formate, ethyl acetate, methyl acetate, ethanol, and formic acid, were analytically pure and obtained from Aladdin (Shanghai, China).

### 4.2. Extraction Process of Lignin from T. arvense Rhizomes

Based on the previous literature and with minor modifications for the extraction process of lignin, a total of 10 g of *T. arvense* rhizome powder was accurately weighed and placed into a 500 mL round-bottom flask, after which 200 mL of 88% formic acid solution was added [56]. The round-bottom flask was placed in a microwave oven and extracted for 30 min. After the extraction process was completed, the mixture was placed in a centrifuge at a speed of 8000 r/min for 10 min, the supernatant was separated into a beaker, the volume of the supernatant was measured, and 5 times the volume of deionized water was added, which was subsequently centrifuged at a speed of 8000 r/min for 10 min to remove the upper layer of solution. The precipitated lignin was washed with deionized water multiple times until the cleaning solution was close to neutral. The extracted lignin was placed in an oven and dried at 60 °C for later use.

### 4.3. Selection of the Lignin Solvent and Antisolvent

**Figure 8:**
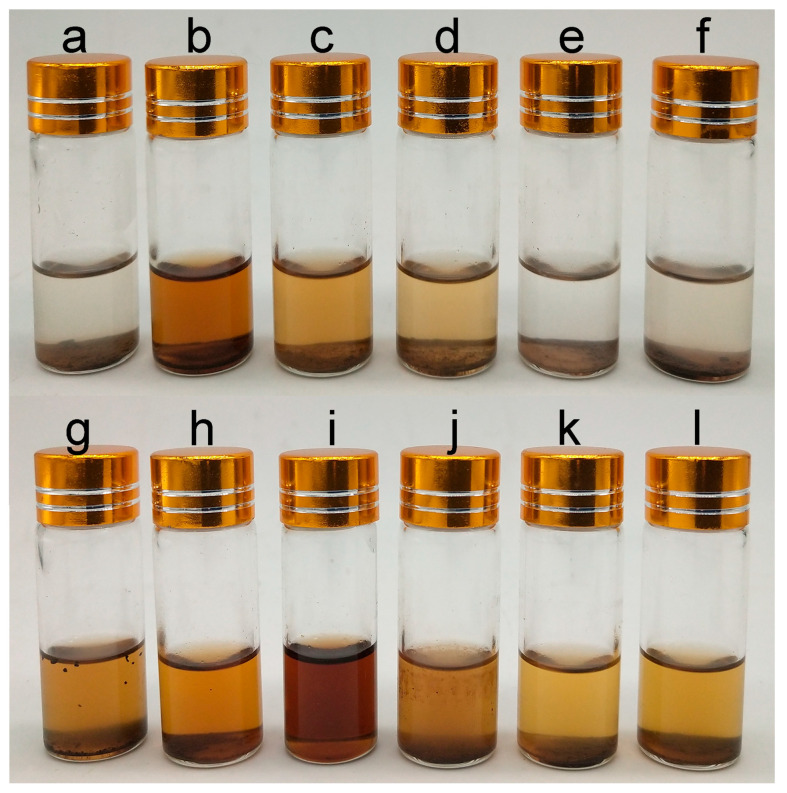
The dissolution of lignin of *Thlaspi arvense* L. rhizomes in different solvents. (**a**). *n*-Butanol, (**b**). tetrahydrofuran, (**c**). ethanol, (**d**). isopropanol, (**e**). *n*-hexane, (**f**). cyclohexane, (**g**). dichloromethane, (**h**). acetone, (**i**). DMSO, (**j**). ethyl formate, (**k**). ethyl acetate, (**l**). methyl acetate.

Lignin powder (20 mg) was added to a glass via l, and 2 mL of *n*-butanol, tetrahydrofuran, ethanol, isopropanol, *n*-hexane, cyclohexane, dichloromethane, acetone, DMSO, ethyl formate, ethyl acetate, or methyl acetate was added. The dissolution of lignin in the same solvent was measured at 280 nm, and photos were taken for recording. Reagents that can completely dissolve lignin as solvents and solvents that cannot dissolve lignin as antisolvents were chosen. The dissolution of lignin by different reagents is shown in Figure 8. On the basis of the solubility analysis results, dimethyl sulfoxide was selected as the solvent, and *n*-butanol was selected as the antisolvent.

### 4.4. Ultrasound-Assisted Antisolvent Precipitation of Lignin Nanoparticles

Twenty milliliters of antisolvent was added to the reaction device, the reaction device was started, and lignin solutions of different concentrations were dripped into the antisolvent at a set rate. Lignin nanoparticles were prepared under the combined action of KQ-250DB ultrasonication (Kunshan, China), mechanical stirring, and shearing. The KQ-250DB ultrasonication device was equipped with variable frequency (45, 80, and 100 kHz) and power (100, 150, 200, and 250 W), and the temperature of the ultrasonic bath was maintained by continuously pumping in and out thermostatic water. After the process was completed, the lignin nanoparticle solution was centrifuged at a speed of 1000 r/min for 10 min, and the prepared lignin nanoparticles were collected and freeze-dried to obtain the finished product. The effects of different factors, including lignin droplet acceleration (1, 2, 3, 4, 5, 6, and 7 mL/min), the lignin concentration (1, 2, 3, 4, and 5 mg/mL), the antisolvent-to-solvent ratio (2, 4, 6, 8, and 10 *v*/*v*), the precipitation temperature (5, 10, 15, 20, and 25 °C), the stirring speed (300, 600, 900, and 1200 r/min), the ultrasound irradiation time (2.5, 5, 7.5, 12.5, and 15 min), and the ultrasound irradiation power (100, 150, 200, and 250 W), on the average particle size (APS) of the lignin nanoparticles were examined to determine the factors and their ranges for the subsequent Plackett–Burman design (PBD) and Box–Behnken design (BBD) optimization experiments.

### 4.5. Experimental Optimization Design

#### 4.5.1. Plackett–Burman Design

PBD is an important factor screening experimental design method that uses fewer experiments to evaluate the significant impact of multiple factors on response values. This experiment used 12 experimental combinations to investigate the effects of 7 factors (dropping speed *X*_1_, lignin concentration *X*_2_, antisolvent-to-solvent ratio *X*_3_, precipitation temperature *X*_4_, stirring speed *X*_5_, ultrasound irradiation time *X*_6_, and ultrasound irradiation power *X*_7_) on the APS of the lignin nanoparticles via the ultrasound-assisted antisolvent precipitation method. On the basis of the results of the single-factor experiment, two test levels were selected for each factor: a low level (−1) and a high level (+1). Each experiment was repeated three times, with the APS of the lignin nanoparticles as the response value. Statistical analysis of the results was conducted via the Design Expert 8 experimental design software, and significance was analyzed via Student’s test.

#### 4.5.2. Box—Behnken Design

First, single-factor and PBD experiments were conducted on the seven factors that affect the APS of the lignin nanoparticles, and then, BBD was used to further optimize the three significant factors selected. The significant factors and ranges selected by PBD were the stirring speed *X*_5_ (600–1200 r/min), the dropping speed *X*_1_ (4–6 mL/min), and the ultrasound irradiation power *X*_7_ (150–250 W). The response values of the APS of the lignin nanoparticles were used to further optimize the influencing factors via 3-factor–3-level BBD to obtain nanolignin with an ideal particle size. Each experiment was repeated three times. A total of 17 randomized experiments were conducted to investigate the independent and interdependent effects of the three factors.

### 4.6. Characterization of Lignin Physicochemical Properties

#### 4.6.1. Scanning Electron Microscopy

The morphology of the prepared lignin nanoparticles was scanned via a JSM-7500F scanning electron microscope (Tokyo, Japan). This microscope has soft beam and R filter functions, with a magnification range of 25–1,000,000 and an acceleration voltage of 0.1–30 kV. The sample was treated by gold spraying and measured under high-vacuum conditions with an acceleration voltage of 20 kV.

#### 4.6.2. Fourier Transform Infrared Spectroscopy

A total of 200 mg of potassium bromide and 2 mg of sample powder were weighed and mixed evenly. The samples were ground thoroughly in a mortar, and then the mixed powder was placed into a mold and pressed for 8 min. Spectral scanning of the sample was performed via a Fourier transform infrared spectrometer. The scanning wavelength range was 4000–400 cm^−1^.

#### 4.6.3. X-Ray Diffraction Analysis

The crystallinity of each sample was measured via X-ray diffraction. The main technical specifications are as follows: scanning range 2*θ*: 5–60°, maximum power: 2 kW, rated voltage: 20–60 kV, rated current: 12–50 mA, and scanning speed: 5°/min.

#### 4.6.4. Thermogravimetric Analysis

A 2–10 mg sample was weighed into the crucible so that it covered the bottom of the crucible, which was balanced, and the thermogravimetric analyzer program was set. The temperature range for measurement was 40–600 °C.

#### 4.6.5. Solubility Test

A total of 1 g of lignin powder was weighed before and after nanomaterialization into a beaker, and 1 L of ultrapure water was added for solubility testing at 37 ± 1 °C and 100 r/min. Five milliliters of lignin solution was added at different dissolution time points for measurement, and the same volume of ultrapure water was added to the beaker until the last sample was removed. The extracted samples were filtered and measured at 280 nm via a UV spectrophotometer.

#### 4.6.6. Determination of Antioxidant Activity

An experimental study on the ability of the lignin samples to scavenge DPPH (2,2-diphenyl-1-pyridylhydrazide) free radicals before and after the nanomaterialization of the Polygonatum sibiricum rhizome was carried out with reference to published methods and with minor modifications [57]. Lignin samples (0.1 mL) with different concentrations were mixed before and after nanomaterialization in dioxane/water (90:10, *v*/*v*) with 3.9 mL of 24 μg/mL DPPH methanol solution. The absorbance of the mixture was measured at 520 nm after reacting at 25 °C for 20 min in the dark. Each concentration of DPPH was measured in triplicate, with the commercial antioxidant BHT used as a reference. The DPPH radical scavenging ability (RSA) of the lignin samples was calculated via the following equation:

$$RSA\;\left(\%\right)=\frac{A_{0}-A_{1}}{A_{0}}\times 100\%$$

where A_0_ is the absorbance of the blank control at 520 nm, and A_1_ is the absorbance of the lignin sample solutions of different concentrations prepared at 520 nm. On the basis of the RSA (%) at different sample concentrations, the half-maximal inhibitory concentration (*IC*_50_) was further calculated.

## Figures and Tables

**Table 1:** The average particle size (APS) of lignin nanoparticles using different levels of Plackett–Burman design extraction variables.

No.	*X*_1_ ^a^	*X*_2_	*X*_3_	*X*_4_	*X*_5_	*X*_6_	*X*_7_	APS (nm)
1	4	3	8	5	600	12.5	250	145
2	6	3	6	15	1200	12.5	150	143
3	6	3	6	5	600	12.5	150	161
4	6	1	8	5	1200	7.5	150	148
5	4	3	8	15	60	7.5	150	153
6	b	1	6	5	1200	12.5	250	153
7	4	1	8	15	1200	12.5	150	142
8	6	1	6	15	600	7.5	250	199
9	6	1	8	15	600	12.5	250	168
10	4	1	6	5	600	7.5	150	175
11	4	3	6	15	1200	7.5	250	141
12	6	3	8	5	1200	7.5	250	135

^a^ *X*_1_: dropping speed, mL/min; *X*_2_: lignin concentration, mg/mL; *X*_3_: antisolvent-to-solvent ratio, *v*/*v*; *X*_4_: precipitation temperature, °C; *X*_5_: stirring speed, r/min; *X*_6_: ultrasound irradiation time, min; *X*_7_: ultrasound irradiation power.

**Table 2:** Analysis of variance and regression analysis of PBD data for the prediction of significant extraction variables on the nanolignin average particle size (NPS).

Source	Sum of Squares	Degree of Freedom	Mean Square	*F*-Value	*p*-Value	Inference
Model	3586.15	7	512.31	20.85	0.0054	*
Residual	98.30	4	24.57			
Cor total	3684.45	11				
Regression data						
Term ^a^	Effect	Coefficient	Standard error	*F*-value	*p*-value	
*X*_1_	−17.90	−8.95	1.43	39.12	0.0033	*
*X*_2_	7.89	3.92	1.43	7.49	0.0522	
*X*_3_	3.30	1.65	1.43	1.33	0.3131	
*X*_4_	5.24	2.62	1.43	3.34	0.1415	
*X*_5_	−23.46	−11.73	1.43	67.23	0.0012	*
*X*_6_	−6.30	−3.15	1.43	4.85	0.0925	
*X*_7_	−13.60	−6.80	1.43	22.58	0.0090	*

^a^ *X*_1_: dropping speed, mL/min; *X*_2_: lignin concentration, mg/mL; *X*_3_: antisolvent-to-solvent ratio, *v*/*v*; *X*_4_: precipitation temperature, °C; *X*_5_: stirring speed, r/min; *X*_6_: ultrasound irradiation time, min; *X*_7_: ultrasound irradiation power; * significant at *p* < 0.05.

**Table 3:** Box–Behnken design with experimental and predicted values for the average particle size (APS) of lignin nanoparticles.

No.	*X*_1_: Dropping Speed (mL/min)	*X*_5_: Stirring Speed (r/min)	*X*_7_: Ultrasound Irradiation Power (W)	APS (nm)	
				Predicted Value	Actual Value
1	4	900	150	178.3	179.6
2	4	600	200	170.0	169.1
3	6	900	250	123.5	122.2
4	5	900	200	135.4	134.4
5	5	600	250	143.5	144.9
6	5	900	200	133.7	134.4
7	5	900	200	132.5	134.4
8	4	1200	200	145.0	145.2
9	5	900	200	135.5	134.4
10	6	600	200	142.3	142.1
11	5	600	150	179.8	179.4
12	6	900	150	148.0	148.6
13	5	1200	250	124.6	125.0
14	5	1200	150	157.7	156.2
15	4	900	250	141.0	140.4
16	5	900	200	134.9	134.4
17	5	1200	200	122.0	122.9

**Table 4:** Analysis of variance (ANOVA) for response surface quadratic model and fit statistics for response values.

ANOVA								
Source	Sum of Squares		Degree of Freedom		Mean Square		*F*-Value	*p*-Value
Model ^a^	5081.78		9		564.64		230.57	<0.0001 *
*X*_1_	1212.78		1		1212.78		495.23	<0.0001 *
*X*_5_	930.96		1		930.96		380.15	<0.0001 *
*X*_7_	2151.68		1		2151.68		878.62	<0.0001 *
*X*_1_ *X*_5_	5.52		1		5.52		2.26	0.1769
*X*_1_ *X*_7_	40.96		1		40.96		16.73	0.0046 *
*X*_5_ *X*_7_	2.56		1		2.56		1.05	0.3406
*X*_1_^2^	47.61		1		47.61		19.44	0.0031 *
*X*_5_^2^	210.02		1		210.02		85.76	<0.0001 *
*X* ^2^	415.81		1		415.81		169.79	<0.0001 *
Residual	17.14		7		2.45			
Lack of fit	10.58		3		3.53		2.15	0.2366
Pure error	6.56		4		1.64			
Corrected total	5098.92		16					
Credibility analysis of the regression equations								
Index mark	Standard deviation	Mean	CV %	Press	*R* ^2^	Adjust *R*^2^	Predicted *R*^2^	Adequacy precision
APS ^b^	1.56	143.98	1.09	179.57	0.9966	0.9923	0.9648	47.845

^a^ *X*_1_: dropping speed, mL/min; *X*_5_: stirring speed, r/min; *X*_7_: ultrasound irradiation power, W; ^b^ APS: average particle size (APS) of lignin nanoparticles, nm. * significant at *p* < 0.05.

## Data Availability

The original contributions presented in this study are included in the article/Supplementary Material. Further inquiries can be directed to the corresponding author(s).

## Supplementary Materials

The following supporting information can be downloaded at: https://www.mdpi.com/article/10.3390/molecules30204070/s1, Figure S1 Three diagnostic plots for model adequacy survey for APS of lignin nanoparticles. Normal plot of residuals (a), the actual responses versus the predicted responses (b) and internally Studentized residuals versus run number (c).
