# Supplementary material for: Preparation of Lignin Nanoparticles from Thlaspi arvense L. Rhizomes via Ultrasound-Assisted Antisolvent Precipitation: Nanostructural Characterization and Evaluation of Their Radical Scavenging Activity
Source: Molecules. 2025 Oct 13;30(20):4070. doi: 10.3390/molecules30204070 (PMC12566317; doi:10.3390/molecules30204070)
Supplement: Supplementary file 1 [file molecules-30-04070-s001.zip › molecules-3857224-supplementary.pdf]

Preparation of lignin nanoparticles from *Thlaspi arvense* L. rhizomes via ultrasound-assisted antisolvent precipitation: nanostructural characterization and evaluation of their radical scavenging activity

Ru Zhao[a,b], Wenjun Xu[a], Yuxiang Tang[a], Jinwen Liu[a], Xiaoli Li[a], Liangui Tan[a], Ailing Ben[a], Tingli Liu[a*], Lei Yang[b*]

[a] Nanjing Engineering Research Center for Peanut Genetic Engineering Breeding and Industrialization, School of Food Science, Nanjing Xiaozhuang University, Nanjing 211171, China

[b] College of Chemistry, Chemical Engineering and Resource Utilization, Key Laboratory of Forest Plant Ecology, Ministry of Education, Northeast Forestry University, Harbin, 150040, China

* Corresponding author E-mail: liutingli@njxzc.edu.cn; yanglei@nefu.edu.cn

2.4. Model adequacy survey

Model adequacy was measured by some diagnostic plots. Three diagnostic plots, namely the actual responses versus the predicted responses, the normal plot of residuals and internally Studentized residuals versus run number, are shown in Figure S1. Figure S1a shows that all reasonably aligned points were distributed and rotated around a straight line, which showed ac high degree of matching between the actual and predicted values obtained by the model [44]. As we can see from Figure S1b, all points in the normal plot of residuals were close to contact with the straight line, meaning that the model of lig-nin nanoparticles was stable and accurate and complied with a normal distribution. The plot of internally Studentized residuals versus run number was employed as shown in Figure S1c. All data points exhibited a random scatter distribution within certain limits (±3), which showed a good fit of the responses to the developed model.

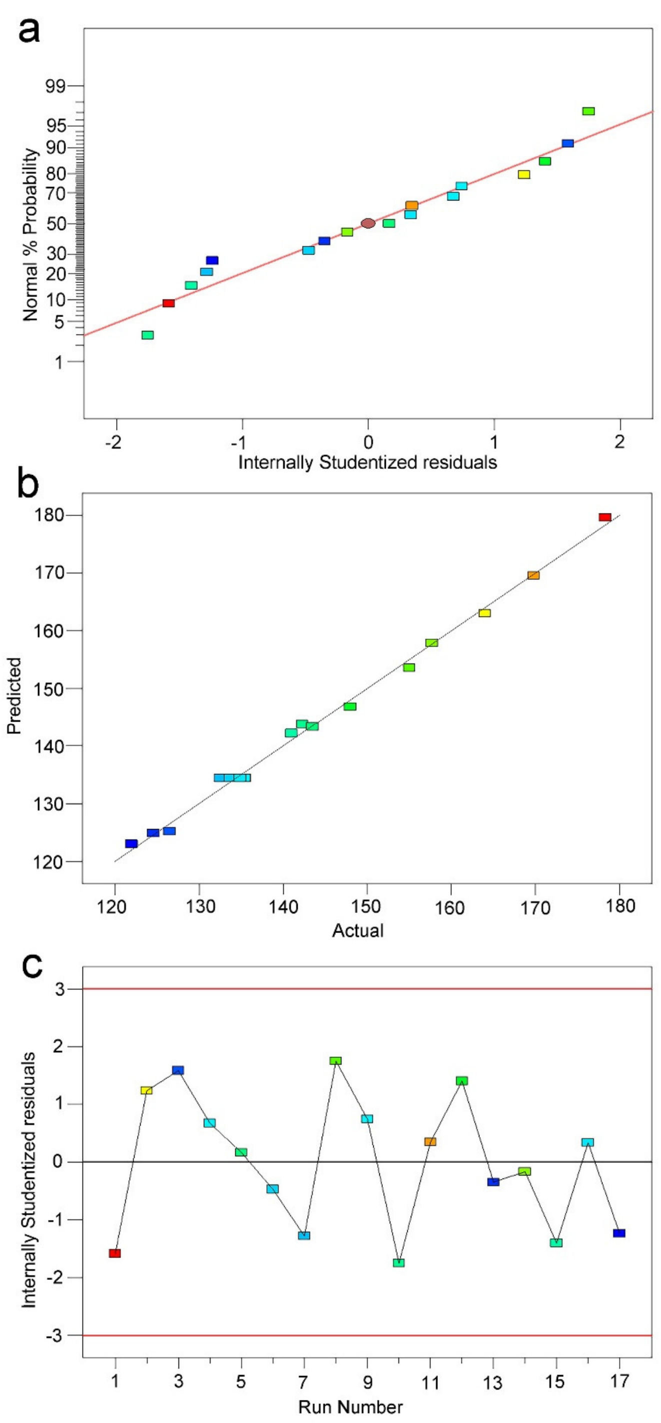

Figure S1. Three diagnostic plots for model adequacy survey for APS of lignin nanoparticles. Normal plot of residuals (a), the actual responses versus the predicted responses (b) and internally Studentized residuals versus run number (c).
